# Supplementary material for: Chromothripsis during telomere crisis is independent of NHEJ, and consistent with a replicative origin
Source: Genome Res. 2019 May;29(5):737–49. doi: 10.1101/gr.240705.118 (PMC6499312; doi:10.1101/gr.240705.118)
Supplement: Supplemental Material [file supp_gr.240705.118_Supplemental_file_1.zip › contigs/annotated_contigs/DB89/contig.3.DB89_length_852_mean_cov_14.2992957746.docx]

**DB89_length_852_mean_cov_14.2992957746**

```
TTCCTCACTGGAGCAGGACTCACTGCTCCACTCTCTGATCCAAAAGGCAGAACCCCTCCCAGCCCCCAAAAAGGCACTGATTGGCCTAA
 >chr2:70374267-70374480 - E=3e-116
CAGAGGGGGAGACATTTTTGGTAAGAGAGGTACTATGCCTCTAAAAGAGAAGGAATAACCACATTGTGTTGTCTACCCTCTGGGATTGA

GGGGCTGGTTTGTGTCAGCTCCTAAGTGCGT|CAGA|GCCAACACACAGAGAAATGCAGAAATGAAAAACCAAAAACCACTCCAACTGC
                                >chr2:70550053-70550360 - E=9e-173
CCTGGAGTCCTTTGGTTTGGCCAAAGCCAGCTCTGTCTCTGCTCTGTCTTTGATTTGATGAGTTACACCAATAAATTCCTTTATTAATT

TCACTTAGTCAGTGTTGGATTTATTAAACCTGGGACTCATCTTTTTCCTTGAAGGCCTAAAAGAACTTCAGCATGTTTTTATCTTGGAT

TCATTTATCCTGATACTGATGCTGCAATCCCTGATTTCTTTGGGTCTGCCTTTGCAAGGCTGCCCTCTTATTT|TG|CTCTACTAAAAA
                                                                            >chr2:70503
TACAAAAAATTAGCCGAGCGCGGTGGCAGGCGCCTATAGTCCCAGCTACTCGGGACGCTGAGGCAGGCGCCTATAGTCCCAGCTACTCG
181-70503526 - E=3e-175
GGACGCTGAGGCAGGAGAATGGCGTGAACCCGGGAGGCGGAGCTTGCAGTGAGCCGAGATAGGCCACTACAGTCCGGCCTGGGCAAAAG

AGCGAGACTCCATCTCAAAAAAAAAAAGATTATTTTTCAAATCCAAGGAGTGATGCCACAGCACTTAACTACACTGGGGAATTTTTTTT

TTTTTTAGATGGAGTCTCACTCCGTCACCCAGGCTAGAGTGCAGTGGTGTGATCT
```
